# Spontaneous and repeat spontaneous abortion risk in relation to occupational characteristics among working Korean women: a cross-sectional analysis of nationally representative data from Korea

**DOI:** 10.1186/s12889-019-7728-7

**Published:** 2019-10-22

**Authors:** Wanhyung Lee, Sung Won Jung, Young-Mee Lim, Kyung-Jae Lee, June-Hee Lee

**Affiliations:** 1 Department of Occupational and Environmental Medicine, Gil Medical Center, Gachon University College of Medicine, Incheon, Republic of Korea; 2 Department of Occupational & Environmental Medicine, Soonchunhyang University Hospital, Seoul, Republic of Korea; 3 Department of Obstetrics and Gynecology, College of Medicine, Ewha Woman’s University, Seoul, Republic of Korea

**Keywords:** Spontaneous abortion, Working women, Korean, Occupation

## Abstract

**Background:**

The association between spontaneous abortion (SA) and occupational characteristics among working women is not well-studied. This study aimed to assess the risk of SA and occupational factors such as occupational classification, working hours, and work schedules among working Korean women aged > 19 years.

**Methods:**

In this cross-sectional study, 4078 working women were identified from among 25,534 workers in the Korea National Health and Nutrition Examination Surveys V (2010–2012) database, to obtain data on SA history and the number of SAs. Odds ratios (ORs) and 95% confidence intervals (CIs) for SA were calculated using multiple logistic regression models after adjusting for age, education, household income, cigarette smoking, alcohol consumption, and obesity status. The weighted prevalence for the number of SAs was calculated according to occupational characteristics to demonstrate the SA status among working Korean women.

**Results:**

SA occurrence was reported in 5.7% of the study participants. The ORs (95% CIs) for SA were significantly higher in pink-, green-, and blue-collared workers than in white-collared workers. Regarding weekly working hours, compared with ≤50 h spent working, the ORs (95% CIs) for 51–60, 61–70, and > 70 h per week were 1.26 (0.87–1.84), 1.63 (1.04–2.56), and 1.73 (1.10–2.70), respectively. A significantly higher weighted prevalence of repeat SAs was observed in pink- and green-collared workers and in those who worked long hours.

**Conclusion:**

We found a significant association between SA, repeat SA, and occupational characteristics among working Korean women.

## Background

Spontaneous abortion (SA), also known as miscarriage, is defined as non-induced embryonic or fetal death, or as an involuntary termination of pregnancy before 28 weeks of gestation [1]. Approximately between 6 and 20% of clinically diagnosed pregnancies may eventuate in an SA, which is the most common adverse pregnancy outcome, owing to multiple factors [2, 3]. Although the major biomechanisms of unwanted early pregnancy termination remain unknown, most SAs are linked to genetic abnormalities or maternal factors including age, health, and behavioral or medical issues [4, 5]. Socioeconomic status (SES) is known to be a key health determinant among women [6], and studies that have focused on SES relating to fertility and childbearing have demonstrated the importance of SES among pregnant women [7–9]. For instance, a low income and educational level may result in adverse pregnancy outcomes owing to a higher likelihood of exposure to risk-taking behavior and hazardous circumstances, and lower access to healthcare services [10, 11].

However, available data regarding the impact of SES-related occupational characteristics on pregnancy outcomes, particularly SAs, is scarce. Certain studies have focused on differences in employment [7] or hazardous factors within the workplace [12], while other studies have indicated that working long hours and working in shifts may be risk factors for adverse pregnancy outcomes [13, 14]. Although there is a growing body of literature that recognizes the considerable impact of occupational characteristics on pregnancy outcomes, to the best of our knowledge, no study has evaluated the association between occupational characteristics such as occupational classification, working hours, or work schedules in South Korea and SA. It is therefore essential to investigate the association between SA and SES with particular focus on occupational characteristics, as this is an area of unmet need in the field of women’s health.

This study aimed to investigate both, the status of SA among working Korean women and the risk factors for SA, including repeat SA, according to occupational classification, working hours, and work schedules, using data from the nationally representative Korea National Health and Nutrition Examination Surveys (KNHANES) database.

## Methods

### Data source

Data were obtained from the KNHANES V (2010–2012) surveys. The KNHANES are a series of cross-sectional, nationally representative, population-based surveys on the health and nutritional status of Korean citizens, that are conducted by the Korea Centers for Disease Control and Prevention (KCDCP) [15]. A total of 25,534 participants (8959 in 2010, 8518 in 2011, and 8057 in 2012) were involved in the KNHANES V, derived from systematic sampling of 100,000 Korean citizens using multistage clusters based on age, sex, and household registries each year. Trained interviewers administered questionnaires on socioeconomic characteristics, health-related behavior, and medical history, including sex-specific health.

**Fig. 1:**
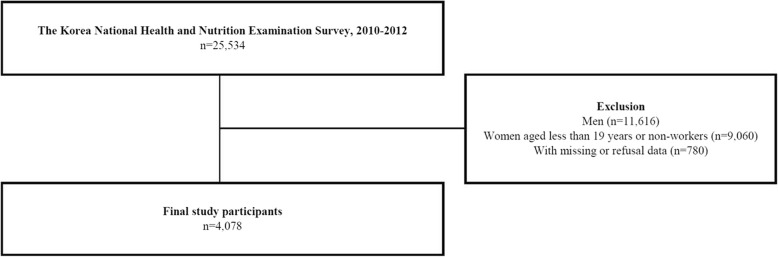
Schematic diagram depicting study population

The current study investigated 4078 working Korean women, and those aged < 19 years, unemployed, or with missing data were excluded. Those who declined to provide data were also excluded. This study was approved by the Institutional Review Board (IRB) of the KCDCP (IRB: 2010-02CON-21-C, 2011-02CON-06-C, and 2012-01EXP-01-2C) (Fig. 1).

### Outcomes

In the KNHANES, participants were surveyed on factors associated with obstetrics and gynecology. The survey was conducted via face-to face interviews by trained KNHANES staff, based on a self-reported questionnaire that included questions regarding a history of SA and the number of SAs previously experienced. SA status was categorized as non-SA (no history of SA), SA (1 SA), or repeat SA (≥2 SAs).

### Covariates

Occupational characteristics were categorized into 3 groups based on 10 major categories as outlined in the International Standard Classifications of Occupations, namely, white-collar workers (managers, professionals, technicians, and associated professionals), pink- and green-collar workers (clerical support, service, and sales workers, and skilled agricultural, forestry, and fishery workers, respectively), and blue-collar workers (crafts and related trades, plant and machine operators and assemblers, and elementary occupations) [16].

To assess the total number of working hours per week, the self-reported questionnaire included the following question: “on average, excluding meal times, how many hours do you work at your job per week including overtime? ” In Korea, the Labor Standards Act, 2018 limited working hours in most workers to 40 h per week, with an additional 12 h of overtime weekly, with the agreement of all parties. During the KNHANES V, the Labor Standards Act had allowed up to 68 working hours per week, and unlimited hours depending on agreement of the worker. Similar to a previous study, to investigate whether the association between weekly working hours and SA was time-dependent, working hours were categorized as ≤50 h, 51–60 h, 61–70 h, and > 70 h [17].

Working schedules were binomially classified according to the responses to the self-reported questionnaire. Those who usually worked during the daytime (06:00–18:00), evening hours (14:00–24:00), or night-time (21:00–08:00) were classified as having fixed schedules, while those who worked according to any other schedule (24-h shifts, split shifts, or irregular shifts) were classified as shift schedule workers.

In addition to the above, SES and lifestyle factors likely to be related to an SA were also investigated. Additional covariates were age (19–40, 41–60, and ≥ 60 years), educational level (middle or lower school, high school, or college level and higher), household income quartile, cigarette smoking (none, past, or current), alcohol consumption (none, standard, or heavy), and body mass index (underweight, normal, overweight, or obese).

The household income level was estimated using standard methods (monthly household income/√of the number of family members) based on sex, residence, and age groups; this was compared to the standard income level of the average Korean household. The adjusted income results were used to categorize quartile levels of household income from lowest (quartile 1) to highest (quartile 4). Past smokers included past smokers who had ceased tobacco smoking for at least 1 month. Those who had smoked fewer than 100 cigarettes in their lifetime were placed in the ‘none’ category. Standard alcohol consumption was defined as the consumption of < 5 and < 7 glasses of alcohol ≤2 times per week for female and male individuals, respectively. ‘Heavy’ alcohol consumption was classified as the consumption of more than the ‘standard’ volume of alcohol. Body mass index (BMI) was categorized into 3 groups based on Asian population standards: underweight (BMI, < 18.5), normal (BMI, 18.5–< 25), overweight (BMI, 25–< 30), and obese (BMI, ≥30). Further details with regard to covariates or survey design are available at https://knhanes.cdc.go.kr/knhanes/eng/index.do.

### Statistical analysis

Data were analyzed using the SAS 9.4 software (SAS Institute Inc., Cary, NC, USA) package. First, the demographics of the study population and the prevalence of SA were described. Chi-squared tests were used to test the significance of the differences in the baseline characteristics (age at the time of survey participation, educational level, household income, cigarette smoking, alcohol consumption, body mass index, occupational classification, work schedule, and weekly working hours) according to SA. ORs and 95% CIs were then estimated to assess the relationship between SA and occupational characteristics using both, crude and adjusted multivariable logistic regression models, for age, educational level, household income, cigarette smoking, alcohol consumption, and obesity status. An adjusted multivariable logistic regression model was condtructed using backward stepwise regression. To determine the SA status of working Korean women, weighted prevalence and standard errors (SEs), based on the number of SAs and occupational characteristics, were calculated using a sample clustering weighting that estimated a complex sampling design and an approximation of the South Korean participants from the KNHANES. For all statistical calculations, a two-tailed *p*-value of < 0.05 was considered statistically significant.

## Results

Among 25,534 workers, we analyzed the data of 4078 working women aged > 19 years, of whom 234 (5.7%) had experienced an SA. There were significant differences in variables such as age at the time of survey participation, occupational classification, educational level, body mass index, and weekly working hours (*p* < 0.05). Among working women who had experienced an SA, 7.3% had graduated from middle school, and 6.9% were secondary school graduates. University graduates demonstrated the lowest rate of SA (2.6%). According to occupational classification, 7.1 and 3.2% of working women in the pink-, green-, and blue-collared and white-collared groups, respectively, reported having an SA. Women who worked > 70 h per week reported a higher percentage of SA (9.8%), followed by those who worked 61–70 h (9.1%), with the lowest SA percentages found in those working for ≤50 h (Table 1).

**Table 1:** Baseline characteristics of study participants according to spontaneous abortion among 4078 of Korean workers aged more than 19 years

	Spontaneous abortion					
	No, (n, %)		Yes, (n, %)		χ^2^	*P*-value
Total subjects	3844	94.3	234	5.7		
Age at survey participation					14.62	0.0001
19–40	1341	96.8	44	3.2		
41–60	1783	92.7	141	7.3		
≥ 60	720	93.6	49	6.4		
Education					25.61	<.0001
Middle school	1406	92.7	110	7.3		
High school	1222	93.1	91	6.9		
College or more	1216	97.4	33	2.6		
Household income					0.06	0.8021
1st Quartile	524	93.4	37	6.6		
2nd Quartile	958	94.6	54	5.3		
3rd Quartile	1143	94.5	67	5.5		
4th Quartile	1219	94.1	76	5.9		
Smoking					0.15	0.6946
None	3431	94.2	211	5.8		
Past	183	94.8	10	5.2		
Current	230	94.7	13	5.4		
Drinking					0.13	0.7124
None	1125	94.3	68	5.7		
Standard	2529	94.1	158	5.9		
Severe	190	96.0	8	4.0		
Body weight					6.15	0.0131
Under weight	236	96.7	8	3.3		
Normal	2536	94.6	146	5.4		
Overweight	916	93.2	67	6.8		
Obesity	156	92.3	13	7.7		
Occupational classification					19.35	<.0001
White-collared	1350	96.8	44	3.2		
Pink/Green-collared	1632	92.9	124	7.1		
Blue-collared	862	92.9	66	7.1		
Work schedule					0.94	0.3317
Fixed	3230	94.4	191	5.6		
Shift	614	93.5	43	6.5		
Weekly working hours					9.08	<.0001
≤ 50	2823	95.1	145	4.9		
51–60	532	93.3	38	6.7		
61–70	250	90.9	25	9.1		
> 70	239	90.2	26	9.8		

**Fig. 2:**
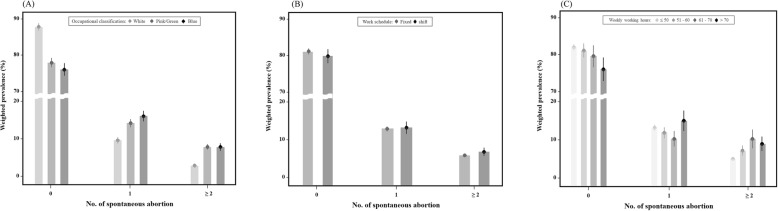
Weighted prevalence of recurrent spontaneous abortion according to occupational characteristics ((**a**) occupational classification, (**b**) work schedule, and (**c**) weekly working hours)

Figure 2 shows the results of the weighted prevalence in regard to the number of SAs according to occupational characteristics, namely, occupational classification, work schedule, and weekly working hours. Concerning occupational classification, Fig. 2 shows that white-collared working women had the highest prevalence rate in the group without SA, whereas blue-collared working women had the highest prevalence rate in the group of working women who had experienced ≥2 SAs. In terms of work schedules, the prevalence rate for fixed working women was slightly higher than shift workers in the group without SA, according to the work schedule; however, shift working women had a slightly higher prevalence rate than fixed working women in the ≥2 SA group. For working hours, those women who worked ≤50 h had the highest prevalence rate in the group without SA, and women working ≤50 h showed the lowest prevalence rate in the group with ≥2 SAs.

Considering the white-collared group as the reference category, the ORs for SA were 1.76 (95% CI: 1.15–2.78) and 1.81 (95% CI: 1.13–3.04) for the pink/green- and blue-collared groups, respectively. Using the fixed hours group as the reference, the OR for the shift working women was 1.27 (95% CI: 0.93–1.80), and considering the group working < 50 h as the reference, the OR was 1.08 (95% CI: 0.72–1.62) for women who worked 51–60 h. However, the OR for women who worked 61–70 h was high, at 1.656 (95% CI: 1.02–2.45), while the risk in women working > 70 h was 1.66 (95% CI; 1.07–2.56) (Table 2).

**Table 2:** Crude and adjusted multivariable logistic regression model of association between occupational characteristics and spontaneous abortion

	Spontaneous abortion			
	Crude model, OR (95% CI)	*p*-value	Adjusted model, OR (95% CI)	*p*-value
Occupational classification				
White-collared	Reference		Reference	
Pink/Green-collared	2.33 (1.64–3.31)	<.0001	1.76 (1.15–2.78)	0.0115
Blue-collared	2.35 (1.59–3.47)	<.0001	1.81 (1.13–3.04)	0.0159
Work schedule				
Fixed	Reference		Reference	
Shift	1.28		1.27 (0.93–1.80)	0.1843
Weekly working hours				
≤ 50	Reference		Reference	
51–60	1.18 (0.79–1.77)	0.4138	1.08 (0.72–1.62)	0.7077
61–70	1.88 (1.21–2.93)	0.0051	1.56 (1.02–2.45)	0.0426
> 70	2.05 (1.32–3.17)	0.0013	1.66 (1.07–2.59)	0.0250

Adjusted model is adjusted for age, education, household income, smoking, drinking, and obesity status

## Discussion

Our findings demonstrate a significantly increased risk of SA among pink-, green-, and blue-collared workers or among women working long hours, after adjusting for SES and health-related behavioral factors. The significant associations between SA and occupational classification and working hours did not attenuate on further analysis using weighted values in working women with repeat SAs. These results suggest that SA and repeat SAs are closely linked to occupational factors related to SES.

The prevalence rates for SA were significantly higher among pink/green-collared and blue-collared working women than in the white-collared working women. These results may be attributed to differing physical demands at work. White-collared workers have less physically demanding work than other occupational categories [18]. Previous prospective studies have attempted to confirm an association between various physical exertion measurements and SA; however, a significant increase in risk was only observed in those who had been standing at work for > 7 h per day [19]. Lifting heavy weights may also be considered to be a risk factor during pregnancy. One study involving working Korean women reported that the risk of SA increased regardless of the number of times they had lifted weights > 5 kg [20]. In a prospective Swedish cohort study, the risk of SA also increased when workers lifted > 12 kg weights more than 50 times per week [21]. Another study indicated a higher risk of SA among workers who were required to maintain an unstable posture while working, such as in a crouching position [22]. Several mechanisms have been suggested to explain the possible adverse influence of physical demands at work during pregnancy on the fetus. The blood flow to the uterus and placenta was found to be reduced in workers who performed heavy physical work, that possibly led to a lower oxygen and nutrient supply to the fetus [23]. Furthermore, reports suggest that increased intra-abdominal pressures owing to lifting, and an unstable posture such as crouching may cause an unwanted early termination of pregnancy [24]. Although the exact weights and unstable postural positions at work have not been specified in the database, it appears that occupational classification differences, including job demands at work, are related to SA.

In previous studies, women with low educational status were also found to be at higher risk of developing adverse pregnancy outcomes compared to the women with a higher education [25, 26]. Our study also demonstrated a high proportion of SA in the low education level group. Women with low education levels are more likely to engage in blue-collar than white-collar jobs [27]; this is supported by our findings.

Some studies have attempted to determine an association between shift work and SA. Although not statistically significant, a meta-analysis reported that the risk of SA increased in a three-shift working schedule (OR = 1.12, 95% CI; 0.96–1.30). However, these findings clearly indicated that the risk increased in those working regularly in night schedules [28]. In the present study, shift work increased the risk of SA; however, the association was not significant. This finding concurs with that of other studies that aimed to determine a correlation between shift work schedules and SA. These studies have consistently shown an increase in the risk of SA. However, not all studies reported statistically significant results [28]. Therefore, it appears that the shift work schedule had a minor effect on SA. However, work schedules are closely linked to circadian rhythm disruption, which may be an adverse risk factor for pregnancy and fetal health [29]. Further studies focusing on work schedules are required.

Till date, a few studies have reported on the effects of long working hours on SA. A systematic review, based on results from developed countries, has shown a significantly increased risk of SA among those working > 40 h per week [28]. Our study also showed a statistically significant association between SA, repeat SAs, and working hours. These results are likely to be related to increased sleep disturbance or stress owing to long working hours [30]. A study has suggested that increased levels of inflammatory serum cytokines may lead to uterine instability, consequently contributing to early termination of pregnancy among working women with short durations and poor quality of sleep [31]. Higher stress among working women with longer working hours has been found to be linked to physiological homeostasis, which may also affect the course of pregnancy. Hypothalamic-pituitary-adrenal activation, as a primary regulator of the stress response, increases stress-related hormone levels, which are known to be a risk factor for SA owing to the vasoconstrictive effect on placental perfusion [32]. Furthermore, long working hours may also be related to unhealthy daily behaviors such as high alcohol consumption and cigarette smoking, that have been reported to be important risk factors for SA [33, 34]. Our study identified the potential risk of working long hours among pregnant working women with respect to adverse pregnancy outcomes such as SA, including repeat SAs. Further research is essential to advance understanding in this important area of unmet need.

This study had certain strengths. It included a nationally representative database to investigate the relationship between occupational factors and SA for the first time, and also provided a research approach that may aid the designing of future studies to examine the causal relationship between occupational factors and SA. However, this study also had some limitations. First, given it was a cross-sectional study, causal relationships could not be identified. However, despite the difficulty in investigating causality, our results confirmed an association between occupational factors and SA [35]. Second, the current findings were obtained from analysis with unmatched survey times and SA, in which relationships between occupational factors and SA remain unclear. Future research based on the time of SA is necessary to reveal any specific cause-and-effect relationship between occupational factors and SA. Third, this study used retrospective data from the KNHANES (2010–2012). Although this dataset may be considered obsolete, national data for evaluating occupational histories and a history of SA among working women are not readily available. Finally, our findings may not be generalized as the study design was based on an ecological approach.

## Conclusions

This study was undertaken to investigate the association between occupational characteristics including occupational classification, work schedule, and long working hours, and SA or repeat SA. Occupational classification and long working hours were found to be related to SA or repeat SA with statistical significance. This study did not find a significant difference between work schedules and SA or repeat SAs. Many countries have refined and implemented laws related to workplace maternal health. However, particularly in Korea, these laws do not provide detailed measures for pregnant working women based on occupational characteristics. It is necessary to provide pregnant working women better protection from possible unwanted terminations of pregnancy by managing their work environments and working conditions more effectively. Further studies in large prospective cohorts are needed to validate our findings.

## Data Availability

KNHANES data are publically available. (https://knhanes.cdc.go.kr/knhanes/main.do).

## Abbreviations

BMI: Body mass index
CI: Confidence interval
IRB: Institutional Review Board
KCDCP: Korea Centers for Disease Control and Prevention
KNHANES: Korea National Health and Nutrition Examination Surveys
OR: Odds ratio
SA: Spontaneous abortion
SE: Standard error
SES: Socioeconomic status
