# Correction to: Spontaneous and repeat spontaneous abortion risk in relation to occupational characteristics among working Korean women: a cross-sectional analysis of nationally representative data from Korea

**DOI:** 10.1186/s12889-019-8034-0

**Published:** 2019-12-16

**Authors:** Wanhyung Lee, Sung Won Jung, Young-Mee Lim, Kyung-Jae Lee, June-Hee Lee

**Affiliations:** 1 Department of Occupational and Environmental Medicine, Gil Medical Center, Gachon University College of Medicine, Incheon, Republic of Korea; 2 Department of Occupational & Environmental Medicine, Soonchunhyang University Hospital, Seoul, Republic of Korea; 3 Department of Obstetrics and Gynecology, College of Medicine, Ewha Woman’s University, Seoul, Republic of Korea

**Correction to: BMC Public Health (2019) 19:1339**

**https://doi.org/10.1186/s12889-019-7728-7**

It was highlighted that the original article [1] contained a mismatch between the result section of the abstract and Table 2. This Correction article shows the incorrect and correct result section of this article’s Abstract.

**Incorrect statement:**

SA occurrence was reported in 5.7% of the study participants. The ORs (95% CIs) for SA were significantly higher in pink-, green-, and blue-collared workers than in white-collared workers. Regarding weekly working hours, compared with ≤50 h spent working, the ORs (95% CIs) for 51–60, 61–70, and > 70 h per week were 1.26 (0.87–1.84), 1.63 (1.04–2.56), and 1.73 (1.10–2.70), respectively. A significantly higher weighted prevalence of repeat SAs was observed in pink- and green-collared workers and in those who worked long hours.

**Correct statement:**

SA occurrence was reported in 5.7% of the study participants. The ORs (95% CIs) for SA were significantly higher in pink-, green-, and blue-collared workers than in white-collared workers. Regarding weekly working hours, compared with ≤50 h spent working, the ORs (95% CIs) for 51–60, 61–70, and > 70 h per week were 1.08 (0.72–1.62), 1.56 (1.02–2.45), and 1.66 (1.07–2.59), respectively. A significantly higher weighted prevalence of repeat SAs was observed in pink- and green-collared workers and in those who worked long hours.
